# Predictive Model for Ambulatory Hypertension Based on Office Blood Pressure in Obese Children

**DOI:** 10.3389/fped.2020.00232

**Published:** 2020-05-19

**Authors:** Girish C. Bhatt, Abhijit P. Pakhare, Priya Gogia, Shikha Jain, Nayan Gupta, Sudhir K. Goel, Rajesh Malik

**Affiliations:** ^1^Department of Pediatrics, All India Institute of Medical Sciences (AIIMS) Bhopal, Bhopal, India; ^2^Department of Community & Family Medicine, All India Institute of Medical Sciences (AIIMS) Bhopal, Bhopal, India; ^3^Department of Biochemistry, All India Institute of Medical Sciences (AIIMS) Bhopal, Bhopal, India; ^4^Department of Radio-diagnosis, All India Institute of Medical Sciences (AIIMS) Bhopal, Bhopal, India

**Keywords:** Ambulatory blood pressure monitoring (ABPM), obesity, white coat hypertension (WCH), ambulatory hypertension, predictive model

## Abstract

**Background:** The epidemic of obesity, along with hypertension (HT) and cardiovascular disease, is a growing contributor to global disease burden. It is postulated that obese children are predisposed to hypertension and subsequent cardiovascular disease in adulthood. Early detection and management of hypertension in these children can significantly modify the course of the disease. However, there is a paucity of studies for the characterization of blood pressure in obese children through ambulatory blood pressure monitoring (ABPM), especially in the developing world. This study aims to characterize ambulatory blood pressure in obese children and to explore feasibility of using office BP that will predict ambulatory hypertension.

**Methods:**In the present study, 55 children with a body mass index (BMI) in the ≥95th percentile for age and sex were enrolled in a tertiary care hospital and underwent 24 h of ABPM and detailed biochemical investigations.

**Results:**Ambulatory hypertension was recorded in 14/55 (25.5%; white coat hypertension in 17/29 (58.6%) and masked hypertension in 2/26 (7.69%). For office SBP percentile the area under curve (AUC) was 0.773 (95% CI: 0.619–0.926, *p* = 0.005) and for office DBP percentile the AUC was 0.802 (95% CI: 0.638–0.966, *p* = 0.002). The estimated cut offs (Youden's index) for office blood pressure which predicts ambulatory hypertension in obese children were the 93rd percentile for systolic BP (sensitivity-67% and specificity−78%) and the 88th percentile for diastolic BP (sensitivity-83% and specificity-62%).

**Conclusion:**Ambulatory blood pressure abnormalities are highly prevalent among children with obesity. Office blood pressure did not accurately predict ambulatory hypertension. More than half of the children labeled as “hypertension” on office blood pressure measurement in the study were diagnosed to have white coat hypertension (WCH), thus emphasizing the role of ABPM for evaluation of WCH before the child is subjected to detailed investigations or started on pharmacotherapy.

## Introduction

The epidemic of obesity, along with hypertension (HT) and cardiovascular disease, is a growing contributor to global disease burden (1). Studies have shown that pediatric hypertension is the strongest predictor of adult hypertension and increases cardiovascular mortality risk in adults (2). Measurement of blood pressure (BP) is difficult in children as there may be variations in BP in response to a number of physiological and environmental stimuli (3, 4). Ambulatory blood pressure monitoring (ABPM) may overcome these challenges and help in characterization of BP in children. The recent American Academy of Pediatrics (AAP) guidelines for screening and management of high blood pressure in children and adolescents included revised normative blood pressure tables based on normal-weight children and excluded overweight and obese children (2). The new guidelines recommends the use of ABPM for confirmation of HT in children, whenever feasible.

Obese children with ABPM have shown a prevalence for ambulatory hypertension ranging from 25% to as high as 48.6% in previous studies (5, 6) with 20% having severe ambulatory hypertension. It has been postulated previously that ambulatory systolic blood pressure and obesity are independently associated with left ventricular hypertrophic remodeling in children (7). However, there is a paucity of studies for the characterization of BP in obese children through ABPM, especially in the developing world. Moreover, ABPM may not always be feasible due to non-availability of the instrument and expertise in interpretation of ABPM data in the pediatric population. The present study aims to characterize ambulatory hypertension on children with obesity and to explore the feasibility of using office BP that will predict ambulatory hypertension as per the recent AAP normative tables.

## Methods

### Study Population and Setting

This is an observational study and was carried out in a tertiary care teaching hospital in central India. As per departmental protocol, all outpatient-registered children undergo anthropometry with a trained staff. For the present study, children aged 5–18 years with a body mass index (BMI) in the ≥95th percentile for age and sex were enrolled after obtaining written informed consent. Children with congenital malformations, chronic kidney disease, congenital heart disease, malignancy or bone marrow transplant, evidence of raised intracranial pressure, taking medications which are known to increase blood pressure, and other systemic illnesses associated with hypertension were excluded from the study. The study was carried out from December 2015 to December 2017.

### Data Collection Procedure and Variables

After obtaining written informed consent, variables such as weight, height, waist circumference, neck thickness, serum uric acid, fasting blood sugar, triglycerides, urinary sodium, and urinary creatinine were collected in a case record form.

### Office Blood Pressure (OBP) and Ambulatory Blood Pressure Measurement Technique

Three resting blood pressure measurements were obtained from the right upper arm using an aneroid sphygmomanometer and appropriate size cuff, on two separate occasions. The office blood pressure was obtained by averaging the systolic and diastolic blood pressure.

For ambulatory blood pressure monitoring (ABPM), a Meditech ABPM-05 device was use (8). The patients underwent 24 h ABPM with an appropriately sized cuff. ABPM was applied to the non-dominant arm and the device was programmed to record BP every 20 min during waking hours and 30–60 min during sleeping hours. After application of ABPM, 3 resting office BP measured with an oscillometric device was compared with the readings obtained through ABPM. Agreement of an average 3 office and 3 ABPM levels within 5 mm Hg was considered appropriate (9). Parents were instructed to maintain a diary of daily activity, sleep, awake time, and medications taken. ABPM variables were reported as mean ambulatory systolic blood pressure (SBP), mean diastolic BP during a 24 h period, daytime and nighttime periods, BP loads (percentage of readings above the ambulatory 95th percentile), and percentage dipping. Ambulatory hypertension was diagnosed when the average ambulatory systolic or diastolic BP was ≥95th percentile for height and sex as per the normative values for ABPM (10). Dipper was classified as having a decrease in average systolic and diastolic BP ≥ 10% during sleep and non-dippers as <10% dipping during sleep (9). White coat hypertension is defined as office BP in the >95th percentile and normal ABPM, while masked hypertension is defined as normal office BP with ambulatory hypertension.

### Laboratory Parameters

Twenty-four hours urinary creatinine and urinary sodium was obtained within 48 h of undergoing ABPM. Serum uric acid, fasting blood sugar, and triglycerides levels were also collected. Salt intake was calculated by using the formula: salt intake = 24 h urinary sodium excretion X (0.0588).

### Statistical Methods

Epi Info 7 (CDC Atlanta) software was used for data entry and analysis. Categorical variables were summarized by frequency and percentage while numerical variables were summarized by mean and standard deviation. Systolic and diastolic blood pressure was measured through ABPM and at office were also converted into categorical variables such as ambulatory hypertension, white coat hypertension, masked hypertension, pre-hypertension, and hypertension by using appropriate cut-offs suggested by AAP. Differences in various clinical and demographic characteristics of those with ambulatory hypertension and those without it were compared by using Chi-square test for categorical variables and *t*-test or Mann-Whitney test for numerical variables. We used Receiver Operating Characteristic (ROC) curve analysis to determine appropriate cut-offs of SBP and DBP office based percentiles against a reference of hypertension diagnosed by ABPM. Optimal cut-off was determined by Youden's index. For ROC analysis we used easy ROC web tool (11).

This study protocol was approved by the Institutional Human Ethics Committee (IHEC) of the institute (IHEC-LOP/2015/IM0080). All children diagnosed as having hypertension were managed as per standard treatment protocols. Nutrition, physical activity, and other lifestyle modifications for obese children were also provided as per standard protocol.

## Results

A total of 55 obese children underwent detailed procedure and investigations. The mean age (SD) of our study population was 11 years (±2.39) with a male to female ratio of 1.8. Family history of hypertension was present in 69% of children. Mean (SD)salt intake in male and female obese children was found to be 8.32 (±3.5) gm and 5.01 (±2.6) gm, respectively (Table 1).

**Table 1:** Distribution of study population by general factors which may be associated with obesity (*n* = 55).

**Variables**	**Mean**	**SD**
Age	11	±2.39
**Gender**		
Male (*n*; %)	36	65.45%
Female (*n*; %)	19	34.55%
Family history HTN (*n*; %)	38	69.09%
Gestational age (weeks)	37	±2.29
Birth weight (kgs)	2.83	±0.52
Height (cms)	143.34	±13.98
Weight (kg)	51.61	±15.57
Waist/Height	0.57	±0.05
Neck/Height	0.21	±0.01
BMI	26.04	±8.47
**BMI percentile (*****n*****; %)**		
Grade 0 (95–97)	15	27.7%
Grade 1 (97–99)	20	36.16
Grade 2 (>99)	20	36.36
Salt intake (g)	7.5	±3.64
Salt intake in males (g)	8.32	±3.5
Salt intake in females (g)	5.01	±2.6

**Figure 1:**
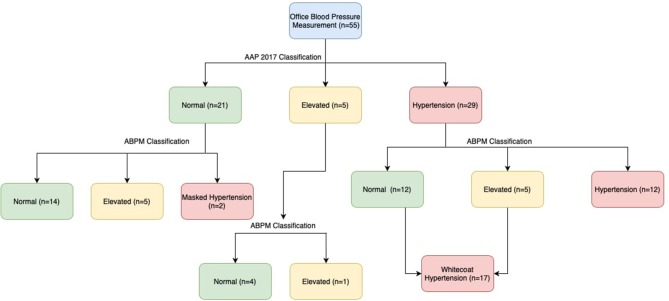
Flow diagram showing the characterization of blood pressure.

As per the new AAP standards, elevated blood pressure (office) was present in 9% and office hypertension in 52.7% of the obese children. Ambulatory hypertension was recorded in 14/55(25.45%), white coat hypertension (WCH) in 17/29 (58.62%), and masked hypertension (MH) in 2/26 (7.69%). Impaired dipping for systolic blood pressure was recorded in 63.6%, while diastolic impaired dipping was recorded in 50.9% of the obese children (Table 2, Figure 1).

**Table 2:** Blood pressure characterization by ABPM & office BP.

**Indicators**	**Mean**	**Standard deviation**
**Office HTN**		
Office SBP	112.38	±10.12
Office DBP	75.0	±7.89
**Office HTN grade (*****n*****;%)**		
Normal	21	38.18%
Pre-HT	5	9.09%
HT	29	52.73%
**ABPM measurements**		
Overall SBP	108.1	±8.47
Overall DBP	64.75	±5.88
Day SBP	110.68	±8.61
Day DBP	67.82	±7.4
Night SBP	102.42	±12.36
Night DBP	59.26	±7.49
Ambulatory dipping	N	%
SBP	35	63.64%
DBP	28	50.91%
**ABPM classification**		
Ambulatory HTN (Overall)	14/55	25.45%
Daytime	2	3.64%
Night time	13	23.64%
White coat HTN among children labeled as Hypertensive (*n* = 29) on Office BP Measurement	17/29	58.62%
Masked HTN among Normal /Elevated (*n* = 26) on Office BP Measurement	2/26	7.69%

The prevalence of ambulatory hypertension increased with increasing BMI (Table 3) and the ratio of waist to height was higher in obese children with ambulatory hypertension (0.58 ± 0.04 vs. 0.54 ± 0.03) however, it was not found to be statistically significant. Day time diastolic hypertension, night time systolic hypertension, and night-time diastolic hypertension was present in 3.6, 12.7, and 21.8%obese children, respectively (Table 4).

**Table 3:** Distribution of variables by stratified by presence of hypertension.

**Factors**	**Hypertension**		**No Hypertension**		***p*-value**
	**Mean (*n*)**	**SD (±)**	**Mean (*n*)**	**SD (±)**	
Weight	57.57	±17.77	49.58	±14.43	0.09
Height	148.21	±15.08	141.68	±13.37	0.0032
BMI	28.14	±10.75	25.32	±7.57	0.286
**BMI percentile (*****n*****;%)**					
Grade 0 (95–97)	2	13.33%	13	86.67%	0.450
Grade 1 (97–99)	6	30.00%	14	70.0%	
Grade 2 (>99)	6	30.00%	14	70.0%	
Neck/Height	0.21	±0.018	0.21	±0.018	0.345
Waist/Height	0.58	±0.04	0.54	±0.05	0.388
FBS	87.97	±6.90	88.39	±8.57	0.875
TG	130.93	±52.45	113.97	±49.82	0.08
Serum Uric acid	5.19	±1.42	5.18	±1.30	0.982
Urinary sodium	137.53	±49.23	116.73	±65.26	0.147
Salt intake in males	8.49	±2.52	8.05	±3.9	0.142
Salt intake in females	5.05	1.89	5.00	±2.8	0.822
SRBD Score	0.2714	0.1817	0.2941	0.1791	0.656

**Table 4:** Ambulatory blood pressure monitoring classification.

**ABPM classiifcation**	**24 h**	**Day-time**	**Night-time**
	***n* (%)**	***n* (%)**	***n* (%)**
**Hypertension**			
Overall	14 (25.5)	2 (3.7)	13 (23.7)
Systolic	7 (12.8)	0 (0)	7 (12.8)
Diastolic	14 (25.5)	2 (3.7)	12 (21.9)
Elevated Blood Pressure	(0)	(0)	(0)
Overall	11 (20)	8 (14.6)	11 (20)
Systolic	11 (20)	8 (14.6)	11 (20)
Diastolic	11 (20)	4 (7.3)	11 (20)

**Figure 2:**
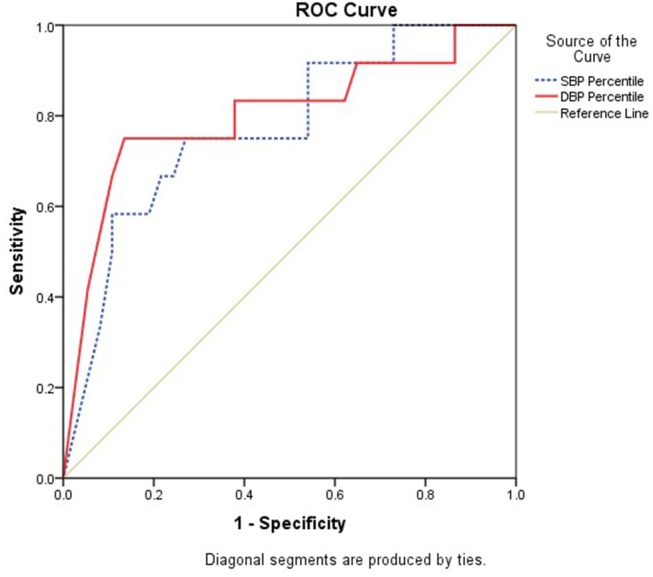
Receiver Operating Characteristic (ROC) Curve analysis to determine appropriate cut-offs of SBP and DBP office based percentiles against reference of hypertension diagnosed by ABPM.

**Figure 3:**
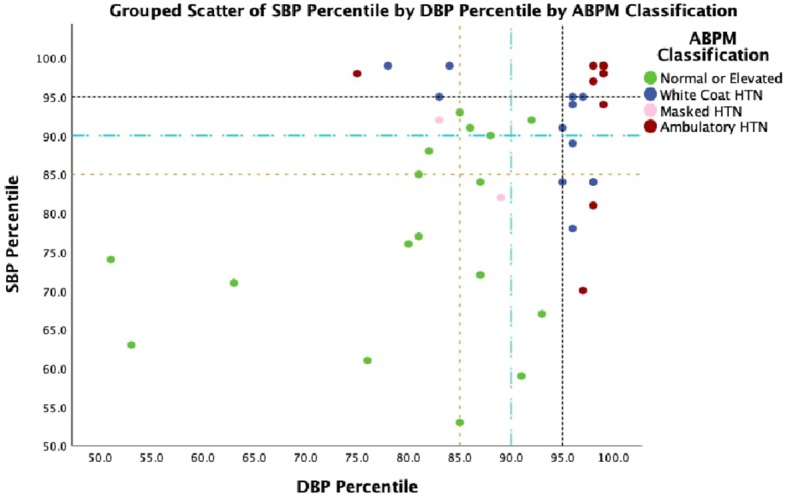
Grouped scatterplot of SBP and DBP percentile showing ABPM classification.

We also calculated the office blood pressure which will predict ambulatory hypertension in obese children as per the recent AAP nomograms. For office SBP percentile the area under curve (AUC) was 0.773 (95% CI: 0.619–0.926, *p* = 0.005) and for office DBP percentile, the AUC was 0.802 (95% CI: 0.638–0.966, *p* = 0.002) (Figure 2). The optimal cut offs for office blood pressure which predicts ambulatory hypertension in obese children were in the 93rd percentile for systolic BP (sensitivity-67% and specificity−78%) and the 88th percentile for diastolic BP (sensitivity−83% and specificity−62%). However, these cut-offs are lower than those used for the definition of hypertension based on readings taken in clinic or office and still have lower sensitivity and specificity, thereby indicating the indispensability of ABPM for the diagnosis of hypertension in children. We assessed various cut-offs of office blood pressure to identify children who have to undergo ABPM for diagnosis of hypertension. Figure 3 shows that, office SBP > the 85th percentile or DBP > the 85th percentile can be used as cut-off for the decision of doing ABPM which will not miss children with ambulatory hypertension.

## Discussion

The present study shows a high prevalence of ambulatory hypertension in obese Indian children (25.45%) with a high percentage of children having impaired dipping status. In the present study, the presence of masked hypertension and white coat hypertension is found to be 7.69 and 58.62% respectively, using the newer AAP nomograms for office blood pressure monitoring. Our estimated cut-offs to predict ambulatory hypertension are lower than the existing definition of hypertension based on office reading and do not have optimal diagnostic test properties, thus emphasizing the role of ABPM for diagnosis of hypertension in obese children. A recent study assessing the impact of 2017 blood pressure guidelines by AAP in overweight/obese youth found a 13% increase in the prevalence of high blood pressure as compared to European Society of Hypertension Guidelines 2016 (12, 13). Another study comparing AAP 2019 normative tables and fourth report found a 12% increase in the prevalence of children with blood pressure in at least the 90th percentile was observed by using the new nomogram without yielding any added advantage in identifying individuals with early cardiac organ damage (13).

The American Academy of Pediatrics 2017 guidelines emphasized on evaluation for white coat hypertension before diagnosing hypertension, as the observed frequency of WCH is high in pediatric studies (14, 15). The prevalence of WCH in our study was more than 58%, implying that these children would have been subjected to unnecessary tests and inappropriate treatment in the absence of ambulatory blood pressure monitoring. The present evidence regarding the underlying mechanism and long term consequences of WCH on cardiovascular health in children remains unclear (14, 16).

Previous studies utilizing ABPM in obese children have shown the prevalence of ambulatory hypertension ranging from 20% to as high as 60% (5, 6, 17). The severity of hypertension increased with an increasing BMI in the present study, which is in concordance with previous studies. Similarly, the white coat effect was more pronounced in obese children, which was postulated in previous studies (18). Another important finding was the presence of a non-dipping pattern in obese children which has been linked to worse cardiovascular outcome (19, 20).

After publications of newer AAP nomograms, only a few studies were conducted to predict ambulatory hypertension based on office blood pressure in adolescents. Hamdani et al. evaluated ambulatory blood pressure monitoring in 247 adolescents and found that clinic systolic in the 85th percentile may be the optimal threshold at which ambulatory BP monitoring should be performed (21). The authors also concluded that 2017 clinical practice guidelines have a higher sensitivity for ambulatory hypertension detection when compared with fourth report percentiles.

The major strengths of the present study are: (1) This study demonstrates feasibility and significance of performing ABPM among obese children. (2) This study also suggests that by using AAP 2017 clinical practice guidelines, a higher number of obese patients are diagnosed with hypertension.

The study is not without limitations, with some major limitations being: (1) The clinical BP was measured by an auscultatory method and ambulatory blood pressure was measured by an oscillatory method, which are not correlated and have restricted reproducibility (21, 22); (2) a formal sample size calculation was not done and convenient sample size based on hospital visits over a duration of 2 years was used. A small sample size in the study was another limitation. (3) Since this study was conducted on children with obesity, the model can't be generalized to children without obesity and lastly, currently used German nomograms are derived from a homogenous Caucasian European population, which is likely not generalizable to children of different races worldwide.

## Conclusion

Ambulatory blood pressure abnormalities are highly prevalent among children with obesity. Office blood pressure did not accurately predict ambulatory hypertension. More than half of the children labeled as “hypertension” on office blood pressure measurement in the study were diagnosed as having white coat hypertension (WCH), thus emphasizing the role of ABPM for the evaluation of WCH before the child is subjected to detailed investigations or started on pharmacotherapy.

## Data Availability Statement

Due to ethical restrictions imposed by the author's Ethics committee, it is not possible to deposit data to a public repository. Interested researchers are kindly asked to send appropriate requests to the corresponding author Girish C. Bhatt, drgcbhatt@gmail.com.

## Ethics Statement

The studies involving human participants were reviewed and approved by All India Institute of Medical Sciences, Bhopal. Written informed consent to participate in this study was provided by the participants' legal guardian/next of kin.

## Author Contributions

GB conceptualized and wrote the manuscript. PG, SJ, SG, NG, and RM helped in carrying out the research work and reviewed the manuscript for critical inputs. AP and GB developed the protocol and carried out statistical analysis. All authors read and approved the final version of the manuscript.

## Conflict of Interest

The authors declare that the research was conducted in the absence of any commercial or financial relationships that could be construed as a potential conflict of interest.
